# Letter to the Editor: Comparison of postoperative glomerular filtration rate between robot-assisted partial nephrectomy (RAPN) and open partial nephrectomy (OPN)

**DOI:** 10.1097/JS9.0000000000001544

**Published:** 2024-05-03

**Authors:** Xuancheng Zhou, Bo Li, Guanhu Yang, Hao Chi

**Affiliations:** a Clinical Medical College, Southwest Medical University; b Department of General Surgery, The Affiliated Hospital of Southwest Medical University; c Academician (Expert) Workstation of Sichuan Province, Metabolic Hepatobiliary and Pancreatic Diseases Key Laboratory of Luzhou City, The Affiliated Hospital of Southwest Medical University, Luzhou, Sichuan, People’s Republic of China; d Department of Specialty Medicine, Ohio University, Athens, Ohio, USA

Renal cell carcinoma (RCC) has the highest mortality rate among genitourinary tumors, but if detected early, and in the few cases where there is a risk of recurrence, RCC can be cured by surgery^1^. Restricted RCC (cancer that is only present in the kidney and has not spread to other organs outside of the kidney) is considered to be an earlier stage of kidney cancer and has a better outcome and prognosis. Partial nephrectomy is preferred for the treatment of limited RCC in the EAU (European Association of Urology) RCC guidelines^2^.

We have noted the meta-analysis conducted by Qu *et al*.^3^, aimed at comparing the therapeutic outcomes of robot-assisted partial nephrectomy (RAPN) and open partial nephrectomy (OPN) for localized RCC. No meta-analysis comparing these two surgical treatment modalities has been done before. The intraoperative blood loss, duration of surgical hospitalization, perioperative complication rate, proportion of patients requiring transfusion during the perioperative period, and glomerular filtration rate (GFR) decline in renal function – these five outcome measures collectively indicate a significant advantage of RAPN over OPN. This will contribute to informed decision-making for clinicians and patients alike.

Among the five significant outcome measures identified by Qu *et al.*, we noted that intraoperative blood loss (*I* ^2^=97%), surgical hospitalization duration (*I* ^2^=59%), and postoperative decline in renal function GFR (*I* ^2^=46%) show moderate to high heterogeneity across studies (*I* ^2^>30%). This suggests less agreement among studies, possibly due to differences in interventions, outcome measurements, and study quality. Particularly, since fewer studies are included for these three measures, each study significantly influences the meta-analysis’s interpretation. Hence, conducting sensitivity analysis is crucial. However, the authors didn’t report results from the sensitivity analysis. To comprehensively assess the study findings, we conducted a sensitivity analysis on three indicators: intraoperative blood loss, surgical hospitalization duration, and postoperative decline in renal function GFR. Table 1 details our sensitivity analysis results, revealing robustness in surgical hospitalization duration outcome but instability in postoperative decline in renal function GFR and intraoperative blood loss outcomes.

**Table 1:** Sensitivity analysis.

GFR after two surgical procedures					
Study of removal	Chanwooo Lee 2016	Hidekazu Tachibana 2019	Sangchul Lee 2021	Sangjun Yoo 2016	
MD	−3.24 [−5.01, −1.47]	−1.70 [−3.99, 0.60]	−1.92 [−3.63, −0.20]	−2.64 [−4.27, −1.01]	
Blood loss					
Study of removal	Hidekazu Tachibana 2019	Ki Don Chang 2013	Sangchul Lee 2021	Toshio Takagi 2016	
SMD	−1.26 [−2.66, 0.14]	−1.29 [−2.60, 0.02]	−2.81[−3.29, −2.33]	−1.12 [−2.14, −0.10]	
Hospitalization time					
Study of removal	Chanwooo Lee 2016	Hidekazu Tachibana 2019	Ki Don Chang 2013	Sangchul Lee 2021	Toshio Takagi 2016
MD	−1.67 [−2.25, −1.09]	−1.44 [−2.14, −0.74]	−1.81 [−2.19, −1.43]	−1.41 [−1.98, −0.84]	−1.62 [−2.15, −1.09]

GFR, glomerular filtration rate; MD, mean difference; SMD, standard mean difference.

We particularly focused on observing the outcome measure of postoperative decline in renal function GFR. After excluding the study by Tachibana *et al*. that was included in the meta-analysis, we found that the difference in the postoperative decline in renal function GFR was not statistically significant. Due to the significant contribution of the study by Tachibana *et al*.^4^, which accounts for a substantial proportion of the sample size (53.8%) in the meta-analysis results; its data greatly influenced the outcome measure of postoperative decline in renal function GFR. Therefore, conducting an in-depth evaluation of the study by Tachibana *et al*. is necessary. We read the study by Tachibana *et al*. with great interest. We found that they presented data on the decline in renal function GFR at 1, 3, and 6 months postoperatively. In the RAPN group, the decline in GFR was 7.1 at 1 month postoperatively and 7.4 at 6 months postoperatively. However, what is perplexing is that the decline in GFR at 3 months postoperatively exhibited a significant difference from both 1 and 6 months postoperatively, with a decrease to 5.7. This indicates a significant instability in the data at 3 months postoperatively. In the meta-analysis conducted by Qu *et al*., the analysis of postoperative decline in renal function GFR focused on the values extracted at 3 months postoperatively. Due to concerns regarding the quality of the data at 3 months postoperatively, we chose to reanalyze the data using the decline in renal function GFR at 6 months postoperatively, which represents long-term stable values. The results did not demonstrate statistical significance (*P*=0.06), and there was a decrease in heterogeneity among the included studies. This also aligns with our previous findings from the sensitivity analysis, where the exclusion of the study by Tachibana *et al*. resulted in similar outcomes. It reiterates that, compared to OPN, RAPN cannot be conclusively proven to effectively reduce postoperative decline in renal function GFR. Tachibana *et al*. also noted in their article that as postoperative time increases, the difference in decline of renal function GFR between the two groups diminishes, providing further support to our viewpoint.

In conclusion, this study conducted a comprehensive statistical analysis comparing RAPN and OPN across multiple outcome measures. While some uncertainty remains regarding whether RAPN effectively reduces postoperative decline in renal function GFR compared to OPN, it doesn’t undermine the main conclusions. RAPN’s superiority is evident across various outcome measures, adding significant value to clinical decision-making.

## Ethical approval

Not applicable.

## Consent

Not applicable.

## Sources of funding

The study was approved by the projects of the Science and Technology Department of Sichuan Province (2023YFQ0101) and the Chen Xiao-Ping Foundation for the Development of Science and Technology of Hubei Province (CXPJJH123003-008).

## Author contribution

X.Z., B.L., G.Y., and H.C.: conceptualization, methodology, validation, formal analysis, investigation, resources, data curation, writing – original draft, writing – review and editing, supervision, and project administration.

## Conflicts of interest disclosure

There are no conflicts of interest.

## Research registration unique identifying number (UIN)

Not applicable.

## Guarantor

Xuancheng Zhou, Bo Li, Guanhu Yang, and Hao Chi.

## Data availability statement

The data used to support the findings of this study are included in the article.

## Provenance and peer review

Not applicable.

## References

[1] Cairns P . Renal cell carcinoma. Cancer Biomark 2010;9:461–473. 22112490 10.3233/CBM-2011-0176 PMC3308682

[2] Ljungberg B Bensalah K Canfield S . EAU guidelines on renal cell carcinoma: 2014 update. Euro Urol 2015;67:913–924. 10.1016/j.eururo.2015.01.005 25616710

[3] Qu H Wang K Hu B . Meta-analysis of clinical outcomes of robot-assisted partial nephrectomy and classical open partial nephrectomy. Int J Surg 2024;Apr 3 [Epub ahead of print]. doi:10.1097/JS9.0000000000001324 PMC11487007 38573087

[4] Tachibana H Kondo T Yoshida K . Lower incidence of postoperative acute kidney injury in robot-assisted partial nephrectomy than in open partial nephrectomy: a propensity score-matched study. J Endourol 2020;34:754–762. 32368924 10.1089/end.2019.0622

